# Effects of Cultivation Conditions and Bean Curd (Tofu) Wastewater Application on ALA Accumulation in *Chlorella* sp. L166 and Its Mutant C-12

**DOI:** 10.3390/foods15091524

**Published:** 2026-04-28

**Authors:** Xiaoxuan Zhou, Shuo Wei, Xuechao Zheng, Ye Chen

**Affiliations:** 1 College of Food Science and Biology, Hebei University of Science and Technology, Shijiazhuang 050018, China; 19903611663@163.com (X.Z.); 15226622137@163.com (S.W.); 2 State Key Laboratory of Food Nutrition and Safety, College of Food Science and Engineering, Tianjin University of Science & Technology, Tianjin 300457, China

**Keywords:** *Chlorella* sp., ALA, accumulation, bean curd (tofu) wastewater

## Abstract

Alpha-linolenic acid (ALA) is an essential omega-3 fatty acid and a vital component in food applications. In this study, we investigated a range of physicochemical culture conditions—including pH, temperature, and carbon source—to evaluate biomass and ALA accumulation in *Chlorella* sp. L166 and its mutant, C-12. The study aimed to identify favorable culture conditions and evaluate the feasibility of using diluted bean curd (tofu) wastewater as a low-cost medium. Under mixotrophic cultivation, ALA content was determined via GC-MS, and the removal efficiencies of total nitrogen (TN), total phosphorus (TP), and chemical oxygen demand (COD) were simultaneously monitored. The results showed that L166 achieved its highest ALA accumulation at pH 6.0 and 23 °C with maltose. C-12 exhibited appropriate ALA accumulation at pH 7.0 and 23 °C with maltose and reached its maximum biomass at pH 8.0 and 25 °C with glucose. After 8 days of cultivation in threefold-diluted tofu wastewater, C-12’s ALA content reached 6.1 mg/g, significantly higher than that observed in BG11 medium. Meanwhile, both strains removed 81.2–83.2% of TN, 35.7–36.0% of TP, and 42.6–43.5% of COD. This study provides preliminary data on the effects of culture conditions on microalgal ALA production, highlighting the potential for future practical applications of C-12.

## 1. Introduction

Alpha-linolenic acid (ALA) is an essential omega-3 fatty acid for the human body; it can reduce blood pressure, blood glucose, and blood lipids, as well as prevent platelet aggregation, thrombosis, and inflammation [1]. At present, ALA is primarily derived from crops such as flaxseed, soybean and rapeseed [2], although some oil crops (e.g., perilla) have an ALA content of 65% to 70% of total fatty acids in their seeds [3]. With the growing global demand for high-quality ALA, these traditional sources are facing bottlenecks including limited arable land and restricted cultivation areas [4], thus struggling to meet the increasing market demand [5]. The development of alternative, high-quality sources is thus vital to meet the human need for ALA [6]. As a novel resource, some microalgae exhibit outstanding ALA content, with their ALA accounting for 20% to 40% of total fatty acids, far higher than that of common oil crops [7], making them a highly promising alternative source to address the aforementioned supply shortage [8].

Microalgae do not compete with food crops for arable land or drinking water, and they exhibit a short growth cycle and strong environmental adaptability, enabling them to survive in various complex environments [9]. Among numerous microalgae species, *Chlorella* sp. benefits from the above advantages, and its ALA content is higher than that of other microalgae strains [10]. Obtaining high-ALA-yielding *Chlorella* species is useful for the production of microalgal-based ALA [11]. Low-temperature plasma (LTP) can be used to mutagenize microalgae [12], and a high-yield *Chlorella* strain (C-12) was obtained via LTP mutagenesis in our previous work [13]. However, the accumulation of ALA in microalgae was significantly influenced by various environmental variables, such as temperature and pH [14]. In particular, pH is one of the most critical environmental conditions in microalgae culture: it determines the solubility and availability of CO_2_ and nutrients, affects microalgal metabolism, and involves algal specificity [15]. Heterotrophic growth is achievable in some microalgae in carbon-containing media such as glucose, fructose and sucrose. This approach benefits from good control, high biomass and fat content, and a low requirement for light. This method also achieves a shorter production cycle and more concentrated product accumulation, further improving production efficiency [16]. Meanwhile, temperature is also an important factor affecting the composition of fatty acids in microalgae [17]. Therefore, we conducted a systematic investigation of the culture conditions for both the wild-type L166 and the mutant C-12 to screen out favorable conditions for biomass and ALA accumulation.

In investigating culture conditions to improve ALA production, reducing production costs is a core prerequisite for the practical application of microalgae-derived ALA [18]. Finding low-cost, highly compatible culture media is key to reducing production costs. As a resource rich in nutrients such as organic carbon, nitrogen, and phosphorus, food-processing wastewater, especially bean curd (tofu) wastewater [19], features low toxicity, and its nutrient composition is well-matched to the growth requirements of some microalgal strains [20]. Certain microalgae can purify wastewater by removing total nitrogen (TN), total phosphorus (TP), and chemical oxygen demand (COD) while utilizing these nutrients for growth [21]. Among them, *Chlorella* sp. has shown a remarkable ability to remove nitrogen, phosphorus and organic matter from food-processing wastewater [22]. However, undiluted tofu wastewater has high turbidity and excessive nutrient concentration, which may inhibit the growth of microalgae, including *Chlorella* sp. [23]. Moreover, the suitability of this wastewater for ALA accumulation in L166 and C-12 strains, including key indicators such as nutrient utilization efficiency and ALA productivity, is yet to be clarified.

In light of the above context, in this study, we focus on *Chlorella* sp. L166 and its mutant, C-12. We systematically investigate three key environmental variables (pH, temperature, and carbon source) to determine the effects of these physicochemical variables on biomass and ALA accumulation in the two *Chlorella* strains. We evaluate their capacity to remove TN, TP, and COD from tofu wastewater and verify the feasibility of the culture medium. Finally, we compare and analyze the performance of L166 and the mutant C-12 under different culture conditions and wastewater systems, evaluating the potential of the mutant strain in ALA production and wastewater resource utilization. The results of this study can provide theoretical support and a technical reference for future scale-up studies assessing *Chlorella* as a functional food platform for ALA production with both environmental and economic benefits.

## 2. Materials and Methods

### 2.1. Algae Strain and Culture Conditions

*Chlorella* sp. L166 was obtained from the Algae Collection of the Applied Microalgae Biology Laboratory of the Ocean University of China (Qingdao, China). C-12 is a high-yield ALA mutant of *Chlorella* sp. L166 induced by LTP in our previous work [13]. All strains were pre-cultured in 200 mL of BG11 medium, contained in 250 mL Erlenmeyer flasks, at a 5% inoculation ratio [24]. Cultivation was performed in a constant temperature incubator at 25 °C. The light intensity was 18 μmol photons m^−2^ s^−1^, and the algae were cultured in a 12 h/12 h light/dark cycle, without additional aeration. It should be noted that this relatively low light intensity resulted in a light-limited condition, leading to a slow phototrophic growth rate. During the exponential growth phase, they were inoculated into the respective experimental conditions with an initial OD_680_ of 0.12 for subsequent experiments.

#### 2.1.1. Determination of OD_680_

The optical density of the *Chlorella* suspension at 680 nm (OD_680_, and therefore has no unit) was measured using a UV-visible spectrophotometer (model: E-201-C-9; Shanghai Luoshu Technology Co., Ltd., Shanghai, China). Before measurement, the culture broth was gently shaken to ensure uniform cell distribution, and BG11 medium was used as the blank control to calibrate the spectrophotometer. All measurements were performed in triplicate.

#### 2.1.2. Calculation of Biomass

A linear relationship was observed between the OD_680_ values and dry cell weight (DCW); thus, the growth curves were created based on OD_680_. To quantify biomass, a linear regression equation between OD_680_ and DCW was established [25]. Briefly, the *Chlorella* suspension was centrifuged at 8000 rpm for 10 min; the supernatant was discarded, and the cell pellet was freeze-dried (FD-10, Beijing De Tianyou Technology Development Co., Ltd., Beijing, China) until reaching a constant weight. The DCW in the sample was calculated according to Formula (1):

(1) $$y=0.2748x+0.00118\left(R^{2}=0.992\right)$$

### 2.2. Determination of the Content of α-Linolenic Acid

*Chlorella* cells in the exponential growth phase were centrifuged at 4000 r/min for 5 min. Sterile water was added to the cell pellet to resuspend the cells. The ALA content of *Chlorella* sp. L166 and C-12 were determined using the sulfuric acid–methanol method. The *Chlorella* cells were freeze-dried; then, 20 mg of dry cells was added to a 10 mL centrifuge tube, followed by 8 mL of the sulfuric acid–methanol (*v*/*v* 10%) solution. Then, the sample was treated with ultrasound (100 W) for 20 min. Water (2 mL) and n-hexane (2 mL) were then added; the n-hexane layer was extracted 1 h later and sealed using a membrane (0.45 μm) to prepare for gas chromatography–mass spectrometry (GC-MS) analysis. To determine the ALA content in line with the external standard method, ALA methyl ester (Shanghai Yuanye Biotechnology Co., Ltd., Shanghai, China) was used as the standard substance. Gradient concentration solutions of ALA methyl ester (0 mg/mL, 5 mg/mL, 10 mg/mL, 15 mg/mL, 20 mg/mL, and 25 mg/mL) were prepared with chromatographic-grade n-hexane and filtered through a membrane for use. The fatty acid composition and ALA content were determined via GC-MS using a GCMS-QP2010 (Shimadzu, Kyoto, Japan) instrument. Helium was used as the carrier gas, with a split flow rate of 7.5 mL/min and a carrier gas flow rate of 1 mL/min. The temperature program was set as follows: the initial temperature was 60 °C, which was held for 5 min; the temperature was then increased to 245 °C at a rate of 10 °C/min, finally being held at 245 °C for 20 min. ALA content (mg/g) was estimated according to the standard curve shown in Formula (2):

(2) $$y=(x+39763.09)/66905.37\left(R^{2}=0.9924\right)$$

### 2.3. Determination of the Content of Total Phosphorus

The TP content was determined as follows: Varying volumes (0.00 mL, 0.25 mL, 0.50 mL, 1.50 mL, 2.50 mL, 5.00 mL, and 7.50 mL) of monopotassium phosphate standard solution were added to a plugged tube. Distilled water was then added separately to a volume of 12.50 mL. Next, 2 mL of potassium persulfate solution was added, and the mixture was shaken until an even consistency was achieved. We then carried out high-temperature and high-pressure digestion. The pressure in the pot reached 1.1 kg/cm^2^ and the temperature reached 120 °C. After 30 min, the digestion process was stopped. When the pressure of the steam sterilizer dropped to 0, we removed the plugged tube, allowing it to cool naturally. Distilled water was then added up to a marked line, after which 500 μL of ascorbic acid was added to the samples. They were allowed to react for 30 s. We next added 1 mL of molybdate solution, which was then kept at room temperature for 15 min. The absorbance of the sample was determined at 700 nm, using the digestion treatment solution of distilled water as a reference. The TP content in the sample was calculated according to Formula (3):

(3) $$y=0.3969x+0.0062\left(R^{2}=0.9994\right)$$

### 2.4. Determination of the Content of Total Nitrogen

The TN content, which includes the sum of water-soluble nitrogen and nitrogen elements in suspended particles, was determined as follows: Varying volumes (0.00 mL, 0.20 mL, 0.50 mL, 1.00 mL, 3.00 mL and 7.00 mL) of potassium nitrate standard solution were added to 25 mL plugged test tubes. We added distilled water, diluting them to 10 mL, followed by 5 mL of alkaline potassium persulfate solution. We covered the tube plug, wrapping it in newspaper and cotton thread to prevent it from bursting out. Next, we placed the plugged tube into a high-pressure steam sterilizer and heated it to 120 °C for 30 min. It was then removed and cooled naturally at room temperature. We gently shook the tube to mix the liquid evenly. Then, 1.0 mL of 10% (*v*/*v*) hydrochloric acid solution was added to each plugged tube, and distilled water was then added up to the marked line. A UV photometer was used to determine the absorbance of the samples at 220 nm and 275 nm, with the distilled water treatment solution used as a blank control. The corrected absorbance was A = A_220_–A_275_. The procedure for determining the TN in the sample was similar: 10 mL of the sample to be tested was added to a plugged tube, and the content of TN in the water sample was calculated according to Formula (4).

(4) $$y=0.0411x+0.0018\left(R^{2}=0.98\right)$$

### 2.5. Determination of the Content of COD

The determination of COD as a measure of water quality was carried out by using a COD determination kit (Guangdong Huankai Microbiology Technology Co., Ltd., Guangzhou, China).

### 2.6. Experimental Design of Culture Conditions of Chlorella

All experiments were performed with three biological replicates (each replicate was an independent culture flask), and the initial inoculation OD_680_ was uniformly set to 0.12.

#### 2.6.1. Effect of pH on α-Linolenic Acid in *Chlorella* sp.

*Chlorella* sp. cells in the exponential growth phase were centrifuged at 4000 rpm for 5 min, and the resulting cell pellet was resuspended in sterilized water. The algal suspension was then inoculated into BG11 medium, and the initial pH was adjusted to 5.0, 6.0, 7.0, 8.0 or 9.0 using HCl or NaOH. Phototrophic cultivation was performed in a constant temperature incubator at 25 °C under a light intensity of 18 μmol photons m^−2^ s^−1^ (a light-limited condition) and a 12 h/12 h light/dark cycle, without additional aeration. The pH was monitored every two days using a pH electrode (PHS-25, INESA Co., Ltd., Shanghai, China) and then readjusted to the original pH using 1 mol/L HCl or 1 mol/L NaOH [26]. After 30 days of cultivation, the biomass of *Chlorella* sp. was measured, and the content of ALA was determined.

#### 2.6.2. Effect of Temperature on α-Linolenic Acid in *Chlorella* sp.

The *Chlorella* sp. cells in the exponential growth phase were centrifuged at 4000 rpm for 5 min, and the resulting cell pellet was resuspended in sterilized water. The algal suspension was then cultivated at 23 °C, 25 °C, 27 °C and 29 °C for 30 days. Phototrophic cultivation was performed in a constant temperature incubator under a light intensity of 18 μmol photons m^−2^ s^−1^ (a light-limited condition) and a 12 h/12 h light/dark cycle, without additional aeration. The content of ALA in cells was determined using the GC-MS method after freeze-drying.

#### 2.6.3. Effect of Carbon Source on α-Linolenic Acid in *Chlorella* sp.

This experiment was conducted under a mixotrophic cultivation mode. *Chlorella* sp. cells in the exponential growth phase were centrifuged at 4000 rpm for 5 min, and the cell pellet was resuspended in sterilized water. The algal suspension was inoculated into 200 mL of BG11 medium (contained within 250 mL Erlenmeyer flasks) supplemented with different carbon sources (glucose, galactose, maltose, or sucrose) prior to sterilization (121 °C, 20 min). The concentrations of the carbon sources were 0.4 mol/L, 0.4 mol/L, 0.2 mol/L, and 0.2 mol/L, respectively, designed with reference to the methods described by Zhan et al. [27] and Kim et al. [28]. During the 48 h cultivation period, the cultures were routinely examined under an optical microscope to monitor cell morphology and check for bacterial contamination; no contamination was observed. The biomass and ALA concentration were measured after 48 h of culture, and *Chlorella* cells were collected and freeze-dried.

### 2.7. Experimental Design for the Removal of TN, TP, and COD from Bean Curd (Tofu) Wastewater

Samples of tofu wastewater were collected from a soybean product processing factory in Tianjin, China, and diluted threefold with sterile water to adjust the nutrient concentration (initial TN: 308.10 μg/mL, TP: 71.79 μg/mL, COD: 280.75 mg/L) to a level suitable for *Chlorella* growth. Three types of culture media were prepared for this experiment, and their compositions are consistent with Table 1: (1) BG11 medium: Complete standard BG11 medium containing all components; (2) BG11- medium (a nitrogen- and phosphorus-depleted modification): BG11 medium with NaNO_3_, KH_2_PO_4_·3H_2_O and CaCl_2_·2H_2_O removed; (3) Threefold-diluted tofu wastewater medium: Only threefold-diluted tofu wastewater without any additional BG11 components.

Each medium (200 mL) was dispensed into 250 mL Erlenmeyer flasks, sterilized at 121 °C for 20 min, and cooled to room temperature before inoculation. *Chlorella* sp. L166 and C-12 in the logarithmic growth phase were inoculated into the three media with an initial biomass of 0.12 g/L. The culture conditions were consistent with the pre-cultivation: 25 °C, 18 μmol photons m^−2^ s^−1^, 12 h/12 h light/dark cycle, cultured in a constant temperature incubator without additional aeration.

At set time points (0, 2, 4, 6, 8 days), 10 mL of algal culture was sampled and centrifuged at 4000 rpm for 5 min. The supernatant was filtered through a 0.45 μm organic filter membrane to remove particulate impurities. The concentrations of TN, TP and COD in the filtrate were determined according to the methods described in Section 2.3, Section 2.4 and Section 2.5, respectively.

### 2.8. Statistical Analysis

All experiments were performed in triplicate, and the data are presented as the mean ± standard deviation (SD). Prior to analysis of variance (ANOVA), the assumptions of normality and homogeneity of variances were assessed using the Shapiro–Wilk test and Levene’s test, respectively. A one-way ANOVA and Duncan’s test were conducted via IBM SPSS Statistics 19.0 (IBM SPSS Inc., Chicago, IL, USA) to evaluate the statistical significance of differences in all measured variables, including biomass (OD_680_), ALA concentration, ALA productivity, and wastewater treatment variables (TN, TP, and COD). The threshold for statistical significance was set at *p* < 0.05.

## 3. Results and Discussion

### 3.1. Effect of Culture Conditions for Chlorella

#### 3.1.1. Effect of pH on α-Linolenic Acid in *Chlorella*

**Figure 1:**
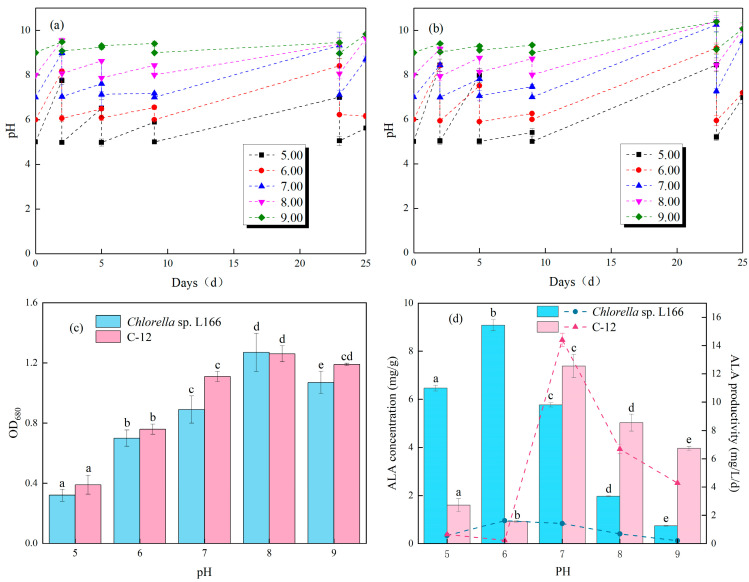
(**a**) pH change for *Chlorella* sp. L166. (**b**) pH change for C-12. (**c**) OD_680_ and (**d**) ALA concentration and productivity. Different letters (a–e) represent significant differences (*p* < 0.05).

We monitored the dynamic changes in medium pH during the cultivation of *Chlorella* sp. L166 and C-12, the results of which are shown in Figure 1a,b. The pH of *Chlorella* sp. L166 and C-12 increased with culture duration under various initial pH levels (5.0, 6.0, 7.0, 8.0, and 9.0), mainly attributed to the absorption of nitrate (NO_3_^−^) and carbon dioxide (CO_2_/H_2_CO_3_) during microalgal metabolism [29]. Nitrate uptake consumes protons (H^+^) to maintain intracellular charge balance, resulting in a pH increase in the medium; CO_2_ addition acidifies the medium and causes a pH decrease, and these two processes balance each other naturally in the nitrate-fed microalgal cultures, which leads to a progressive pH increase during the cultivation [30]. For *Chlorella* sp. L166, initial acidic conditions (pH 5.0–6.0) led to a gradual pH increase, reaching 7.75 and 8.17 after 3 days of cultivation, respectively; neutral to alkaline initial pH levels (7.0–9.0) increased more significantly to 9.00, 9.55, and 9.49. In contrast, C-12 showed a more notable pH rise under acidic initial conditions: the medium pH increased from 5.0 to 8.44 and from 6.0 to 8.45. When the initial pH exceeded 7.0, the pH elevation amplitude of both strains narrowed, and the final pH tended to converge at 9.17–9.55, indicating that alkaline initial conditions weaken the effect of microalgal metabolism on pH [26]. This enhanced acid tolerance of C-12 is closely related to the adaptive response induced by LTP mutagenesis. According to Zheng et al. [13], LTP treatment can induce mutations in stress resistance-related genes of *Chlorella*, which improves the strain’s ability to regulate intracellular pH balance and maintain normal metabolic activity under low-pH conditions [31]. Furthermore, the continuous pH increase observed until the final day of cultivation could be attributed to the ongoing assimilation of NO_3_^−^ and CO_2_. However, during the stationary phase, this metabolic activity is primarily directed toward intracellular lipid and ALA accumulation rather than active cell division [32].

The effect of pH on the biomass accumulation (OD_680_) of the two strains is presented in Figure 1c. Their most suitable culture condition was pH = 8.0, with no significant difference in biomass between L166 (1.27 ± 0.13) and C-12 (1.26 ± 0.05) (*p* > 0.05). Notably, no significant differences in biomass were observed between the two strains at pH 5.0, 6.0, 8.0, or 9.0 (*p* > 0.05); only at pH 7.0 did C-12 (1.12 ± 0.03) show a significantly higher biomass than L166 (0.89 ± 0.09) (*p* < 0.05). This phenomenon of maximum biomass at neutral to slightly alkaline pH is consistent with multiple studies reporting that *Chlorella* sp. KLSc59 achieved maximum biomass at pH 6.0–7.0 [14]. In studies investigating the ideal growth pH of *Chlorella vulgaris* JSC-6, its biomass increased the most in the pH range from 7.0 to 8.0 [33]. The specific growth advantage of C-12 at pH 7.0 is associated with LTP mutagenesis [13]. In contrast, *Chlorella sorokiniana* was reported to grow better at pH < 6.0 [29], reflecting the species-specific pH adaptability of *Chlorella*.

As shown in Figure 1d, pH had a significant impact on both ALA accumulation and productivity, with distinct favorable conditions for the two strains. For L166, the highest ALA concentration (9.1 ± 0.2 mg/g) and productivity (1.62 ± 0.05 mg/L/d) were obtained at pH 6.0, significantly higher than for C-12 (concentration: 0.93 ± 0.03 mg/g; productivity: 0.235 ± 0.013 mg/L/d) under the same condition (*p* < 0.05). C-12 exhibited the maximum ALA concentration (7.4 ± 0.5 mg/g) and productivity (14.4 ± 0.5 mg/L/d) at pH 7.0, while both indices declined sharply beyond this point. Statistical analysis confirmed significant differences in ALA concentration and productivity between L166 and C-12 at all pH levels (5.0–9.0) (*p* < 0.05). This difference in ideal pH for ALA accumulation may be related to the activity of omega-3 fatty acid desaturase (FAD3), the key enzyme catalyzing ALA synthesis [34]. Preechaphonkul et al. found that FAD3 activity in *Chlorella* sp. KLSc59 was highest at pH 6.0, consistent with the ideal pH of L166 [14]. Additionally, pH affects the availability of nutrients such as phosphorus and magnesium [26], which are essential for fatty acid synthesis. Low pH reduces phosphorus solubility, inhibiting lipid synthesis, while neutral pH promotes the absorption of these nutrients to enhance ALA accumulation [35]. This trend contradicts the findings of Qiu et al., who reported that the ALA concentration in *Chlorella sorokiniana* increases with pH elevation [29]. Beyond the differences in optimal pH among species, the intrinsic capacity for ALA synthesis varies significantly across different microalgal strains under near-neutral conditions. For instance, Lin et al. cultivated the ALA-rich *Monoraphidium* sp. strain in standard BG-11 medium for 18 days, recording a maximum ALA productivity of 3.53 mg/L/d [36]. By contrast, the C-12 mutant cultured at a maintained optimal pH of 7.0 in this study achieved a remarkably higher ALA productivity of 14.4 mg/L/d.

#### 3.1.2. Effect of Temperature on α-Linolenic Acid in *Chlorella*

**Figure 2:**
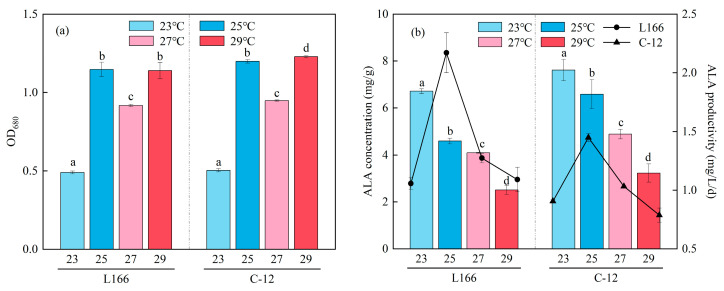
Effect of temperature on the OD_680_ (**a**) and ALA concentration and productivity (**b**) of *Chlorella* sp. L166 and C-12. Different letters (a–d) represent significant differences (*p* < 0.05).

As shown in Figure 2a, after 30 days of cultivation, at 23 °C, the OD_680_ of both strains was significantly lower than that at other temperatures (L166: 0.490 ± 0.009; C-12: 0.504 ± 0.010; *p* < 0.05). At 25 °C, the OD_680_ values of L166 and C-12 reached 1.15 and 1.20, respectively, while the OD_680_ at 27 °C was the second lowest among all tested temperatures (L166: 0.917 ± 0.007; C-12: 0.948 ± 0.004). At 29 °C, C-12 achieved its highest OD_680_ (1.226 ± 0.007), which was significantly higher than that of L166 (1.14 ± 0.05) under the same conditions (*p* < 0.05). No significant difference in OD_680_ was observed between L166 and C-12 at 23 °C, 25 °C or 27 °C (*p* > 0.05), aligning with the findings of Nalley et al. on *Chlorella vulgaris* [37].

Figure 2b presents the variations in ALA content and productivity with temperature. The ALA content in both strains decreased significantly as the temperature increased (*p* < 0.05), consistent with the results of Nalley et al. for *Chlorella vulgaris* [37]. At 23 °C, L166 and C-12 had the highest ALA content of 6.71 ± 0.10 mg/g and 7.6 ± 0.5 mg/g, respectively, the latter being significantly higher than the former (*p* < 0.05). As the temperature increased to 25 °C, 27 °C and 29 °C, the ALA content of L166 decreased to 4.59 ± 0.12 mg/g, 4.10 ± 0.02 mg/g and 2.51 ± 0.19 mg/g, respectively. For C-12, ALA content decreased to 6.6 ± 0.6 mg/g, 4.9 ± 0.2 mg/g and 3.2 ± 0.4 mg/g, respectively. Throughout the temperature range, C-12 maintained a significantly higher ALA content than L166 (*p* < 0.05). L166 achieved the highest ALA productivity at 25 °C (2.17 ± 0.17 mg/L/d), followed by 27 °C (1.27 ± 0.04 mg/L/d); C-12’s highest productivity also occurred at 25 °C (1.44 ± 0.03 mg/L/d). Although 23 °C facilitated the highest ALA content, low biomass at this temperature limited total productivity. In contrast, 25 °C enabled a balance between biomass and ALA content to maximize productivity, which is more conducive to practical application [38]. A high-temperature-induced reduction in ALA content may be ascribed to the following mechanisms: low temperature (23 °C) can induce the activity of ω-3 fatty acid desaturase (FAD3), thereby promoting the conversion of linoleic acid to ALA; high temperature not only inhibits FAD3 activity but also accelerates the β-oxidation of ALA, ultimately resulting in reduced ALA accumulation [39]. This regulatory pattern is also consistent with the observation that polyunsaturated fatty acid content decreases with rising temperature in *Trachydiscus minutus*, further confirming the reliability of the results [40].

#### 3.1.3. Effect of Carbon Source on α-Linolenic Acid in *Chlorella*

**Figure 3:**
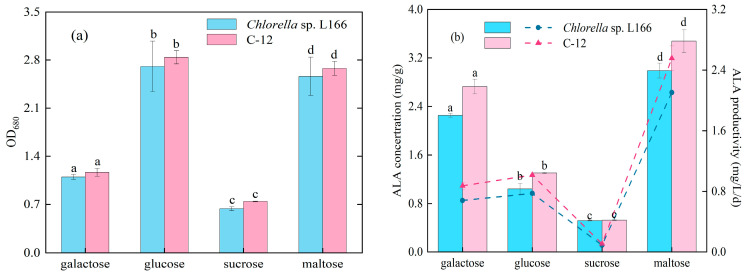
Effect of carbon source on the OD_680_ (**a**) and ALA concentration and productivity (**b**) of *Chlorella* sp. L166 and C-12. Different letters (a–d) represent significant differences (*p* < 0.05).

We investigated various carbon sources by utilizing multiple sugars with the same carbon concentrations. Media were prepared with galactose, glucose, sucrose and maltose at concentrations of 0.4 mol/L, 0.4 mol/L, 0.2 mol/L and 0.2 mol/L, respectively—suitable for *Chlorella* biomass accumulation [27,28]. As shown in Figure 3a, after 48 h of culture, the cells grew rapidly when glucose and maltose were used as carbon sources. *Chlorella* sp. L166 achieved OD_680_ values of 2.7 ± 0.4 and 2.6 ± 0.3 with glucose and maltose, while C-12 reached 2.8 ± 0.1 and 2.68 ± 0.10. This is consistent with the conclusion of Hoang et al. that *Chlorella* exhibited the highest biomass with glucose as the carbon source [41]. C-12 showed higher biomass than L166 with glucose and sucrose, while no obvious difference was observed with galactose or maltose. Glucose was readily metabolized by *Chlorella* cells and rapidly utilized for intracellular metabolic processes, thus contributing to the highest biomass accumulation compared to other carbon sources [42]. Sucrose transport in microalgae relies on a proton co-transport system, and its utilization varies among species [43]. Furthermore, within the short 48 h cultivation period, the high-concentration carbon sources were not completely exhausted due to the unadjusted basal nutrients. Therefore, the differences in OD_680_ primarily reflect the distinct initial assimilation rates of these sugars by the microalgae.

Figure 3b highlights maltose as the most favorable carbon source for ALA accumulation in both strains. *Chlorella* sp. L166 and C-12 displayed ALA concentrations of 2.99 ± 0.12 mg/g and 3.48 ± 0.19 mg/g, respectively—higher than those with other carbon sources. C-12 exhibited a higher ALA concentration than *Chlorella* sp. L166 with other carbon sources. Regarding inter-strain differences, C-12 demonstrated a higher ALA concentration than L166 with galactose, glucose, and maltose, while the two strains showed similar ALA concentrations with sucrose. The ALA productivity showed a positive correlation with its concentration, exhibiting the same pattern of preference for carbon sources. For C-12, the ALA productivity with maltose was significantly higher than that with other carbon sources, reflecting the strain’s strong adaptability to maltose. This preference is closely related to the genetic modification induced by LTP mutagenesis. Our previous transcriptomic analysis of C-12 revealed a 2.47-fold upregulated expression of the gene encoding pyruvate dehydrogenase (E_2_), while that of the gene encoding Acetyl-CoA carboxylase (ACACA) was downregulated by 0.48-fold [13]. This genetic change enhances C-12’s ability to utilize maltose-derived carbon sources—maltose is degraded into glucose and glucose-1-phosphate, which can directly enter the fatty acid synthesis pathway, providing sufficient substrates for ALA synthesis [44]. Meanwhile, upregulated E_2_ activity promotes the conversion of pyruvate to Acetyl-CoA, further supplementing the carbon flux for ALA synthesis, while the downregulation of ACACA reduces the diversion of carbon sources to non-lipid metabolic branches. Such metabolic adjustments collectively position maltose as the most compatible carbon source for C-12, facilitating its efficient accumulation of ALA. In mixotrophic cultivation, the carbon source type impacts both the fatty acid accumulation and the cultivation cycle. Sijil et al. [2] investigated the mixotrophic cultivation of *Desmodesmus* sp. supplemented with a high concentration of glucose (500 mM), which required a 12-day cultivation period to reach a maximum ALA productivity of 4.55 mg/L/d. In this study, the C-12 strain cultivated with maltose (0.2 mol/L) in standard BG11 medium achieved an ALA concentration of 3.48 mg/g within a 48 h period. This comparison suggests that selecting an appropriate carbon source can trigger a rapid metabolic response for ALA synthesis, thereby shortening the necessary cultivation cycle.

### 3.2. α-Linolenic Acid Production and the Removal of TN, TP, and COD by Chlorella *sp.* in Bean Curd (Tofu) Wastewater

#### 3.2.1. α-Linolenic Acid Produced by *Chlorella* sp. in Bean Curd (Tofu) Wastewater

**Figure 4:**
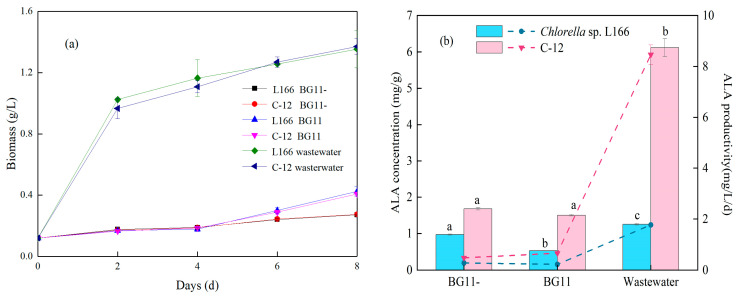
Biomass (**a**) and ALA concentration and productivity (**b**) of *Chlorella* sp. L166 and C-12 in three different culture conditions: BG11 (complete standard BG11 medium), BG11- (BG11 medium without NaNO_3_, KH_2_PO_4_·3H_2_O, and CaCl_2_·2H_2_O), and threefold-diluted bean curd (tofu) wastewater medium. Different letters (a–c) represent significant differences (*p* < 0.05).

As shown in Figure 4a, the biomass of both strains in tofu wastewater (under mixotrophic growth) was significantly higher than that in BG11 and BG11- media (under phototrophic growth) (*p* < 0.05), with no significant difference between L166 and C-12 (*p* > 0.05). Notably, the cultures in the standard BG11 and BG11- media exhibited slow, nearly linear growth. This can be directly explained by the relatively low light intensity (18 μmol photons m^−2^ s^−1^) used in this study, which limited the energy supply for pure phototrophic growth. After 8 days of cultivation, the biomass of L166 and C-12 in wastewater increased from the initial 0.12 g/L to 1.35 g/L and 1.37 g/L, respectively (*p* > 0.05). While phototrophic growth in the standard media was constrained by illumination, the tofu wastewater provided an energy-sufficient mixotrophic environment. This accelerated growth was attributed to the abundant organic carbon, nitrogen, phosphorus, and trace elements in tofu wastewater; the organic carbon acted as an alternative energy source to compensate for the light limitation, fully meeting the nutritional requirements of *Chlorella* growth [45]. Compared with the traditional BG11 medium, the complex nutrient composition of the wastewater promoted the synthesis of cellular components (polysaccharides, proteins, and lipids) through microbial metabolic regulation [46].

Figure 4b shows the ALA concentration and productivity of the two strains in different media. The ALA concentration (6.1 ± 0.2 mg/g) and productivity (8.5 ± 0.4 mg/L/d) of C-12 in tofu wastewater were significantly higher than those of L166 (1.252 ± 0.018 mg/g, 1.77 ± 0.04 mg/L/d, *p* < 0.05), as well as being higher than in BG11 and BG11- medium, indicating that low-temperature plasma (LTP) mutagenesis enhanced the ALA synthesis capacity of C-12 in wastewater. The ALA content and productivity of L166 in bean curd wastewater were also 2.38-fold and 7.96-fold higher, respectively, than those in traditional BG11 medium. It should be noted that the targeted accumulation of specific polyunsaturated fatty acids is strongly influenced by the nutrient profile of the employed wastewater. For instance, Khalaji cultivated *Chlorella vulgaris* in dairy wastewater and reported that the relative proportion of ALA was limited to 1.6–2.2% of the total fatty acids [47]. In the present study, the C-12 strain cultivated in threefold-diluted tofu wastewater achieved an ALA content of 6.1 mg/g and a productivity of 8.5 mg/L/d. The superior ALA accumulation in wastewater was mainly due to the balanced carbon/nitrogen/phosphorus (C/N/P) ratio in wastewater, which improved the activity of ω-3 fatty acid desaturase (FAD3), the key enzyme for ALA synthesis [38]. Additionally, *Chlorella* could efficiently assimilate inorganic nitrogen (NO_3_^−^, NH_4_^+^) and phosphorus in wastewater, converting them into intracellular lipids and fatty acids [18].

#### 3.2.2. Removal of TN, TP, and COD by *Chlorella* sp. in Bean Curd (Tofu) Wastewater

While utilizing nutrients in tofu wastewater to accumulate biomass and ALA, *Chlorella* also exhibits the capacity to purify tofu wastewater, with synergistic removal effects on key pollution indicators including TN (including nitrate nitrogen (NO_3_^−^) and ammonium nitrogen (NH_4_^+^)), TP, and COD [48]. The core mechanism of wastewater purification by algae is biological assimilation: inorganic nitrogen (NO_3_^−^, NH_4_^+^) in wastewater can be directly absorbed by *Chlorella*. However, before utilization, complex organic nitrogen must be degraded into small-molecule nitrogen sources by microorganisms in the system [45]. Phosphorus is mainly taken up by algae in the form of soluble inorganic phosphorus (H_2_PO^4−^ and HPO_4_^2−^) for the synthesis of cellular components such as nucleic acids and phospholipids. Meanwhile, COD is reduced through the synergistic metabolism of algae and microorganisms, converting organic carbon into CO_2_ and biomass [49].

**Figure 5:**
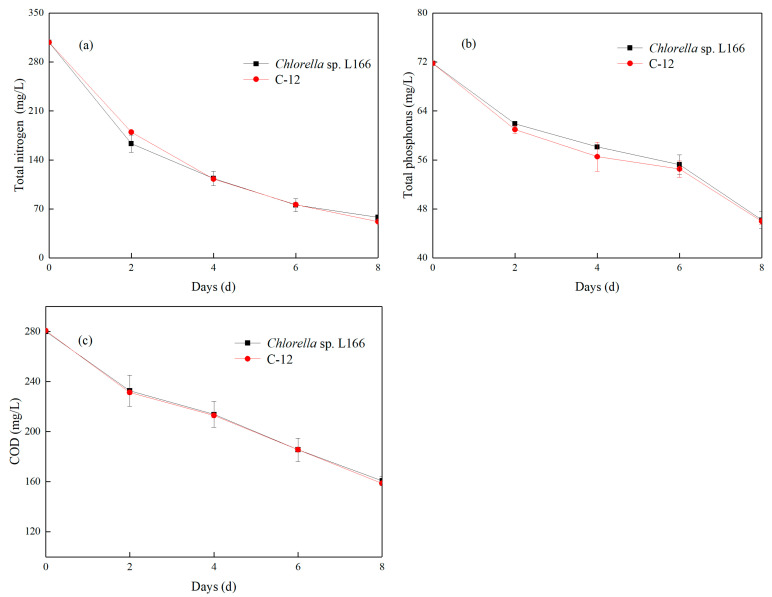
Total nitrogen (**a**), total phosphorus (**b**), and chemical oxygen demand (COD) (**c**) removal ability of *Chlorella* sp. L166 and C-12 in bean curd (tofu) wastewater.

As shown in Figure 5a, inorganic nitrogen was the main nitrogen form in tofu wastewater. The TN removal rates of L166 and C-12 were the highest at 0–2 d and the lowest at 6–8 d of cultivation, with the final removal rates reaching 81.2% and 83.2% at the 8th day; the final TN concentrations decreased to 58 ± 4 μg/mL and 51.83 ± 0.07 μg/mL. This removal efficiency is close to the TN removal rate (93.3%) of *Chlorella* co-cultured with *Phaeodactylum tricornutum* for aquaculture wastewater treatment [50]. It should be noted that the above results were based on lab-scale experiments with a biomass concentration of about 1.5 g/L, which cannot be directly extrapolated to large-scale industrial applications requiring higher biomass concentrations.

Excess phosphorus is a major factor in algal blooms. Effectively removing phosphorus from food wastewater is therefore of great significance [51]. Phosphorus takes multiple forms in wastewater, including soluble, granular, organic and inorganic phosphorus, collectively known as TP. As shown in Figure 5b, after 8 days of cultivation, the TP removal rates of the two strains are 35.7% and 36.0% (*p* > 0.05), with the final TP concentrations decreasing to 46.2 ± 1.4 μg/mL and 45.9 ± 0.5 μg/mL. However, this is lower than that (91.0%) of *Chlorella sorokiniana* in synthetic wastewater [52] but still reflects efficient phosphorus recovery, considering the presence of insoluble organic phosphorus in the bean curd wastewater matrix that requires degradation by microorganisms before utilization.

COD is an important indicator of organic pollution in water. *Chlorella*, *Chroococcus*, *Dunaliella salina*, *Spirulina*, and *Scenedesmus* are commonly used to treat wastewater [53]. As shown in Figure 5c, after two days of inoculation, the COD content in the wastewater decreased sharply; the removal rate of *Chlorella* sp. L166 and C-12 on the eighth day was 42.6% and 43.5%, respectively. The average daily removal amounts of the two *Chlorella* strains were 14.9 mg/L and 15.3 mg/L, respectively. The final COD concentrations decreased to 160 ± 4 mg/L and 159.3 ± 1.2 mg/L, slightly lower than the COD removal rate (58.4%) of *Chlorella vulgaris* in tofu wastewater (40-fold dilution), indicating that microalgae could not utilize all organic carbon in wastewater. Microalgae bioremediation therefore requires additional efforts to replace traditional biological treatment [54].

Comprehensively, the removal efficiency of the various pollution indicators can be ranked as TN > COD > TP. Among these, TN shows the best removal effect (removal rate: 81.2%~83.2%), and its final concentration is below 60 μg/mL.

In this study, C-12 demonstrated significantly enhanced ALA accumulation and volumetric productivity in diluted wastewater compared to the wild type, establishing a promising biological platform for simultaneous wastewater purification and high-value resource recovery. While the current specific ALA content (6.1 mg/g) and overall biomass yield obtained from basic laboratory-scale batch cultivation indicate the need for further enhancement prior to immediate industrial translation, these preliminary findings provide a crucial proof-of-concept. To accurately assess the practical applicability and commercial feasibility of this system, future studies must focus on scale-up validation (e.g., using photobioreactors to improve mass transfer), substantial process optimization, and comprehensive techno-economic assessments.

## 4. Conclusions

This study systematically investigated the effects of key cultivation conditions on biomass and ALA accumulation in *Chlorella* sp. L166 and its mutant, C-12. The latter exhibited a superior ALA accumulation capacity, with the most favorable conditions identified as pH 7.0, 23 °C, and maltose as the carbon source. When cultured in diluted tofu wastewater, C-12 achieved an ALA content of 6.1 mg/g, significantly higher than that in BG11 medium, while both strains efficiently removed 81.2–83.2% of TN, 35.7–36.0% of TP, and 42.6–43.5% of COD from the wastewater. The findings of this study clarify the basic physicochemical variables influencing ALA production in *Chlorella* and the resource potential of tofu wastewater. It should be noted that the current findings are confined to the laboratory level. Nevertheless, the mutant C-12 shows significant promise for simultaneous wastewater treatment and ALA accumulation. Substantial process optimization, scale-up validation, and comprehensive techno-economic assessments are still required to facilitate its commercial application. Overall, this study provides critical baseline data and a fundamental basis for these future investigations.

## Figures and Tables

**Table 1:** Compositions of BG11 medium, BG11- medium and bean curd (tofu) wastewater.

Ingredient	BG11 Medium	BG11- Medium	Bean Curd (Tofu) Wastewater Medium
NaNO_3_	✓ *	- *	-
MgSO_4_·7H_2_O	✓	✓	-
KH_2_PO_4_·3H_2_O	✓	-	-
CaCl_2_·2H_2_O	✓	-	-
Citric acid	✓	✓	-
Ferric ammonium citrate	✓	✓	-
EDTA·2Na	✓	✓	-
Na_2_CO_3_	✓	✓	-
A_5_ *	✓	✓	-
Bean curd (tofu) wastewater	-	-	✓

* A_5_: H_3_BO_3_, MnCl_2_·4H_2_O, ZnSO_4_·7H_2_O, NaMoO_4_·2H_2_O, CuSO_4_·5H_2_O and Co(NO_3_)_2_·6H_2_O. * ✓: indicates the ingredient is included in the medium. * -: indicates the ingredient is omitted.

## Data Availability

The original contributions presented in the study are included in the article, further inquiries can be directed to the corresponding authors.
